# Chromosomal Copy Number Aberrations in Colorectal Metastases Resemble Their Primary Counterparts and Differences Are Typically Non-Recurrent

**DOI:** 10.1371/journal.pone.0086833

**Published:** 2014-02-05

**Authors:** Leonie J. M. Mekenkamp, Josien C. Haan, Daniëlle Israeli, Hendrik F. B. van Essen, Jeroen R. Dijkstra, Patricia van Cleef, Cornelis J. A. Punt, Gerrit A. Meijer, Iris D. Nagtegaal, Bauke Ylstra

**Affiliations:** 1 Department of Pathology, Radboud University Nijmegen Medical Centre, Nijmegen, The Netherlands; 2 Department of Pathology, VU University Medical Center, Amsterdam, The Netherlands; 3 Department of Medical Oncology, Academic Medical Center, Amsterdam, The Netherlands; Duke-National University of Singapore Graduate Medical School, Singapore

## Abstract

The metastatic process is complex and remains a major obstacle in the management of colorectal cancer. To gain a better insight into the pathology of metastasis, we investigated genomic aberrations in a large cohort of matched colorectal cancer primaries and distant metastases from various sites by high resolution array comparative genomic hybridization. In total, 62 primary colorectal cancers, and 68 matched metastases (22 liver, 11 lung, 12 ovary, 12 omentum, and 11 distant lymph nodes) were analyzed. Public datasets were used for validation purposes. Metastases resemble their matched primary tumors in the majority of the patients. This validates the significant overlap in chromosomal aberrations between primary tumors and corresponding metastases observed previously. We observed 15 statistically significant different regions between the primary tumors and their matched metastases, of which only one recurrent event in metastases was observed. We conclude, based on detailed analysis and large independent datasets, that chromosomal copy number aberrations in colorectal metastases resemble their primary counterparts, and differences are typically non-recurrent.

## Introduction

Metastatic disease is the principal event leading to death in patients with colorectal cancer (CRC), yet our understanding of the molecular events leading to metastasis is still incomplete. The formation of metastases is a multistep process, in which malignant cells disseminate from the primary tumor to colonize distant organs [1], [2].

A variety of genetic and epigenetic events that lead to loss of function of tumor suppressor genes, such as *APC*, *TP53* and *SMAD4* and gain of function of oncogenes like *KRAS* and *MYC,* drive tumor cell behavior in a Darwinian selection process. Two hypotheses aim to explain how tumor cells acquire the (epi)genetic alterations that make them proficient to metastasize. The “traditional model” suggests that the metastatic process is accompanied by a sequential accumulation of (epi)genetic alterations [3]. Tumor cells pass through successive rounds of clonal progression and the most malignant cancer cells acquire the capacity to seed new colonies at distant sites [4]. An alternative “predestination” hypothesis, implies that the capacity to metastasize is largely determined by the mutant alleles that are acquired relatively early during tumorigenesis [5]. Subsets of genetic aberrations responsible for oncogenic transformation are also involved in the metastatic progression. This model does not question clonal selection or the accumulation of genetic alterations, but does not place metastatic dissemination near the end of tumor progression [6]. According to this model, primary tumors that can and cannot metastasize will differ more in their biologic features than primary tumors and their associated metastases.

Some studies aimed to unravel metastasis-associated genomic alterations by comparing the genetic profile of metastases with unmatched primary tumors [7], [8]. This approach is of limited value due to the heterogeneity between individuals in the genetic profile of their tumors. There are other studies that use ‘matched’ primaries and metastasis, which however use small datasets [9], [10]. These studies indicated that copy number patterns of metastatic tumor cells are similar to that of the primary tumor. Recurrent copy number aberrations in metastases were not independently validated in large datasets. Since the publication by Stange et al. [9], which reports such a recurrent aberration, the array comparative genomic hybridization (array CGH) technique has dramatically improved. The oligo array CGH technique used here allows for a 20-fold higher spatial detection resolution, with also the capability of detecting important focal aberrations [11]–[15]. In order to improve our understanding of the biology behind the metastatic process, we conducted such high resolution array CGH analysis on a large set of primary CRC and matched metastases of various distant sites.

## Materials and Methods

### Ethics Statement

The two randomized clinical trials, CAIRO and CAIRO2, were approved by the Committee on Human-Related Research Arnhem – Nijmegen and by the local institutional review boards. FFPE tissue of another 8 patients was collected from the tissue archive of the Department of Pathology at the Radboud University Nijmegen Medical Centre, which was approved by the local review board. Approval by the local review boards has been done centrally by Medisch Ethische Toetsingscommissie (METC) Nijmegen. The written informed consent required for all patients before study entry also included translational research on tumor tissue.

### Patients and Tumor Samples

Formalin-fixed paraffin-embedded (FFPE) tissue of surgically resected primary tumor, matched distant metastasis and matched normal colon, was obtained from 62 patients. For 6 patients, tissue samples of two different metastatic sites were collected. Array CGH power analysis shows that this sample size (130 tissue specimens) yields an average power of 0.5 to 0.9 [16]. The 68 metastatic tissue specimens consisted of 22 liver metastases, 11 lung metastases, 12 ovarian metastases, 12 omental metastases, and 11 distant lymph node metastases. The power for these metastatic homing organs is only sufficient to identify the most statistically significant genetic recurrences by array CGH [16].

Eighteen patients included in this study participated in the CAIRO clinical trial [17] (CKTO 2002–07, Clinical Trials.gov; NCT00312000) and 36 patients the CAIRO2 trial [18] (CKTO 2005–02, ClinTrials.gov; NCT00208546) of the Dutch Colorectal Cancer Group (DCCG).

FFPE tissue of another 8 patients was collected from the tissue archive of the Department of Pathology at the Radboud University Nijmegen Medical Centre.

### Clinical and Histopathological Parameters

The following clinical features were collected for each patient: age, gender, site of the primary tumor, metachronous (>6 months after initial diagnosis) or synchronous (≤ 6 months of initial diagnosis) onset of metastases. The TNM classification (5^th^ ed.) [19] was used to describe the extent of cancer spread in terms of invasion depth and lymph node stage. Tumors were histologically classified using the World Health Organization guidelines [20]. A tumor was considered to be of the mucinous type when at least 50% of the tumor volume consisted of mucin. Primary tumors were graded into well, moderately and poorly differentiated adenocarcinomas based on the part of poorest differentiation in the tumor. The mismatch repair system (MMR) status was determined by immunohistochemistry and microsatellite instability (MSI) analysis [21]. Two out of 62 patients (3%) were MMR positive, which is a representative incidence for patients with advanced CRC [21]. Clinical and pathological parameters are summarized in Table 1.

**Table 1:** Baseline characteristics of the 62 patients included in the analysis.

		Patients n, (%) (n = 62)
Gender	Male	33 (53%)
	Female	29 (47%)
Age	Median (range)	60 (34–77)
Site of primary tumour	Colon	29 (47%)
	Rectosigmoid	15 (24%)
	Rectum	16 (26%)
	Unknown	2 (3%)
Onset metastases	Metachronous	30 (48%)
	Synchronous	32 (52%)
Diameter	Median (range)	40 (15–135)
Invasion depth	T1-2	5 (8%)
	T3	47 (76%)
	T4	10 (16%)
Lymph node status	N0	12 (19%)
	N1	22 (35%)
	N2	26 (42%)
	Unknown	2 (3%)
Classification	Adenocarcinoma	54 (87%)
	Mucinous carcinoma	8 (13%)
Differentiation grade	Well	3 (5%)
	Moderate	35 (56%)
	Poor	24 (39%)
MSI status	dMMR	2 (3%)
	pMMR	60 (97%)
Site of metastases	Liver	22 (32%)
	Lung	11 (16%)
	Omental	12 (18%)
	Ovarian	12 (18%)
	Distant lymph node	11 (16%)

Abbreviations: MSI; microsatellite instability, dMMR; deficient mismatch repair system, pMMR; proficient mismatch repair system.

### Chromosomal Copy Number Detection by Array CGH and Data Preprocessing

The procedures for DNA isolation, labeling and hybridization were described previously [22]. DNA was isolated from an area containing at least 70% tumor cells. The 180K CGH arrays (Gene Expression Omnibus (GEO) platform GPL8687 Agilent Technologies, Palo Alto, USA) cover 169.793 unique chromosomal locations across the genome at ∼17 kb intervals, enriched with 4548 additional oligonucleotides, located at 238 of the Cancer Census genes. Array image analysis was performed and local background was subtracted from the signal median intensities of both tumor and normal DNA. The log_2_ tumor to normal ratio was calculated in the statistical programming language R with CGHcall [23] and was normalized against the median value of the log_2_ ratios of all the oligonucleotides mapped to the March 2006 human reference sequence (NCBI36/hg18) on chromosome 1–22 and X.

**Figure 1:**
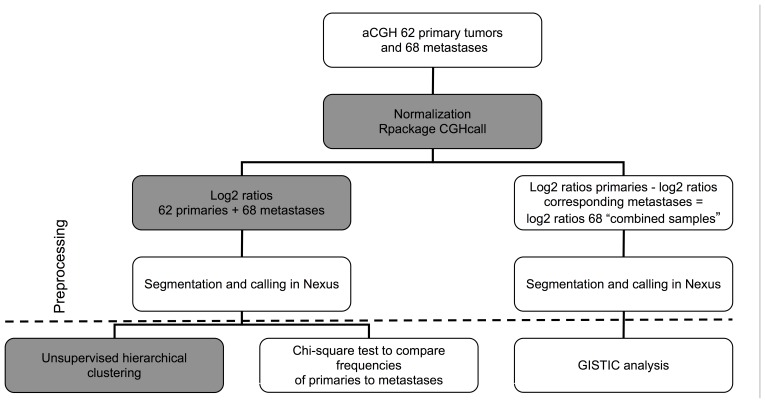
Flowchart of data preprocessing and analysis procedures. In grey analysis performed in R, in white analysis performed in Nexus.

The cellularity parameter in the CGHcall data analysis software was set according to the estimates made by the pathologist (I.D.N.). Further data interpretation and copy number aberration (CNA) calling was done with Nexus Copy Number 6.0 software (Biodiscovery, El Segundo, USA) using default settings, except for the Segmentation Algorithm, which was set to “Rank”. The CNA calling cut-off value for gene copy number gain or loss was set to 0.2 and −0.2, and for amplifications or homozygous deletions this cut-off value was set to 0.6 and −1.0, respectively (Figure 1). The array CGH data can be accessed using GEO, under accession number GSE38479.

### Array CGH Data Analysis

Unsupervised hierarchical clustering analysis was performed in R with the segmented data according to the same procedures as Stange et al. [9]. The distance was calculated based on a Spearman correlation (Figure 1). Copy number concordance of the metastasis with the corresponding primary tumor was defined as a percentage of the genome with the same copy number (gain, loss or normal). Therefore base pair positions of copy number overlap were determined by start and end positions of the segments, which were detected by the segmentation algorithm described above. To compare the tumors pairs, DNA copy number ratios of the primary tumor were subtracted from the corresponding metastases. GISTIC [24] within Nexus 6.0 was used to identify genomic regions that are significantly amplified or deleted across this combined sample set (Figure 1). Output included those regions with a high corresponding G score, indicative of either a high frequency of occurrence or a high amplitude for several samples or a combination. The method accounts for multiple-hypothesis testing using the false-discovery rate (FDR), and a FDR below 0.05 was used as a level of significance.

### Detection of Chromosome 6q21 and 8q24.21 (*MYC*) Co-amplification in Large Independent Cohorts of Primary Colorectal Tumors

The presence of the co-amplification of chromosome 6q21 and 8q24.21 (*MYC*) was assessed in array CGH data of 542 primary colorectal tumors. These array CGH profiles were derived from 349 primary colorectal tumors who participated in either the CAIRO [17] or the CAIRO2 [18] study (JC Haan et al., in preparation), and from 193 primary colorectal tumors present in The Cancer Genome Atlas (TCGA). Chromosomal amplifications were identified by CGHcall [23] for the CAIRO and CAIRO2 samples, and by cBio Cancer Genomics Portal (http://www.cbioportal.org) [25] for the samples of the TCGA dataset, and were only acknowledged if the log_2_ ratio of the segmented values was higher than 2.

### Fluorescence in situ Hybridization (FISH)

Copy number status of both chromosome 6q21 and 8q24.21 (*MYC*), as well as of the centromeres of chromosome 6 and 8 were assessed by FISH analysis [26]. The MYC locus probe (8q24.12-q24.13), 6q21 locus probe (start 106772738–106950984), and the centromere probes of chromosome 6 (6p11.1-q11) and 8 (8p11.1-q11.1) (Vysis, Abbott Molecular, Abbott Park, Illinois, USA). Signals for each probe were counted in at least 40 cells per tumor sample. Samples with a ratio greater than 3 between *MYC* or 6q21 versus the centromere signals, in 10% of cells or more, were scored positive for amplification.

## Results

### Striking Similarity in DNA Copy Number Status between Primaries and Matched Metastases

**Figure 2:**
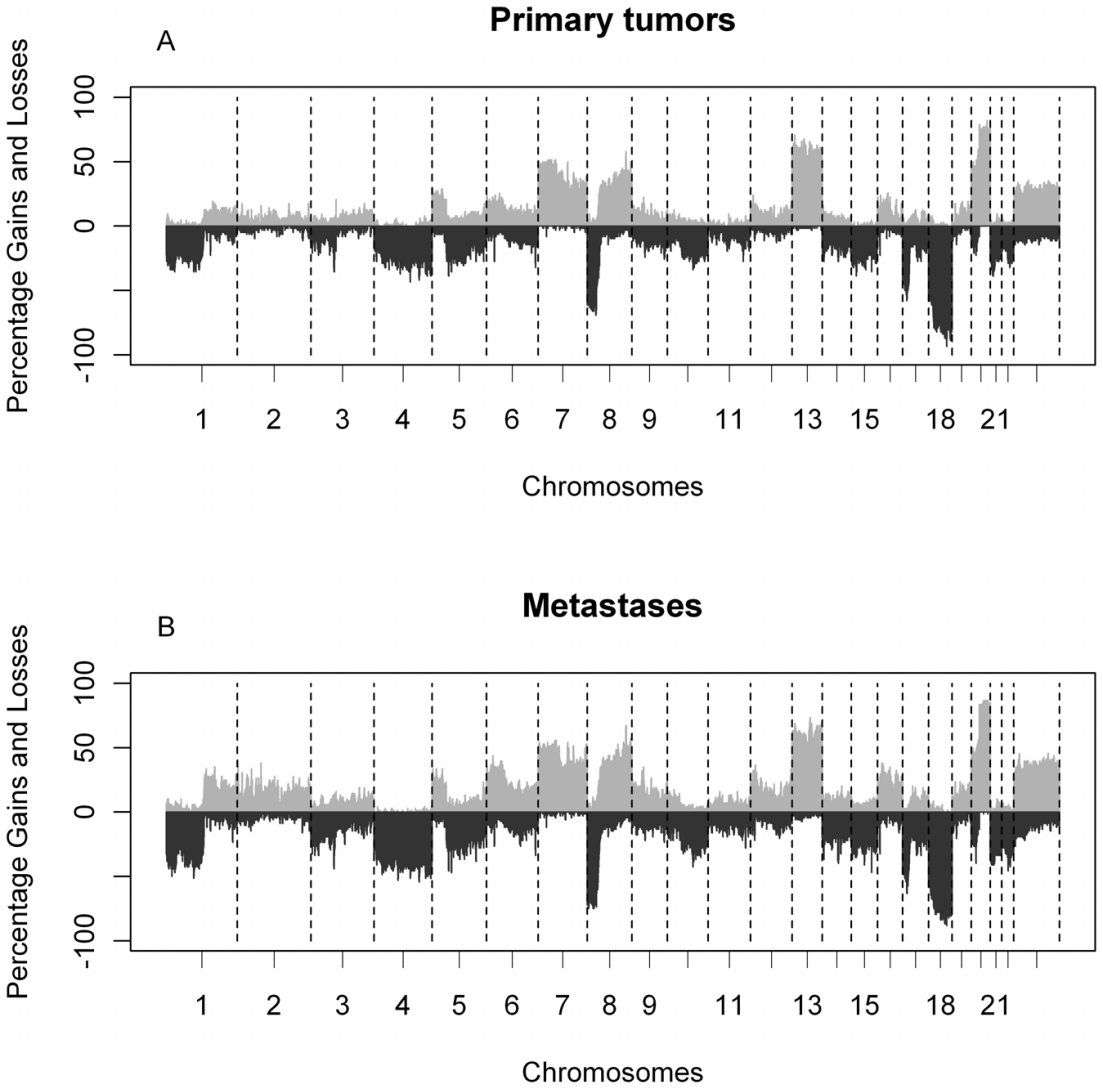
Frequency plots of DNA copy number aberrations in 62 primary tumors and 68 matched metastases. (*A*) Frequencies of aberrations based on called data for primary tumors and (*B*) metastases. The x-axis displays clones spotted on the array sorted by chromosomal position. The y-axis displays the frequency of tumors with gains (above zero) or losses (below zero). Boundaries of chromosomes are indicated by dotted lines.

Patterns of DNA copy number aberrations between 62 primary tumors and 68 matched metastases were highly similar for the majority of the patients (Figure 2). When the group of primaries was compared to the group of metastases, only gain of chromosomes 2p25.3 and 2q21.3 were more frequently observed in metastases (p<0.001; Table S1). However, after correction for multiple testing no significant regions were left (FDR>0.05).

**Figure 3:**
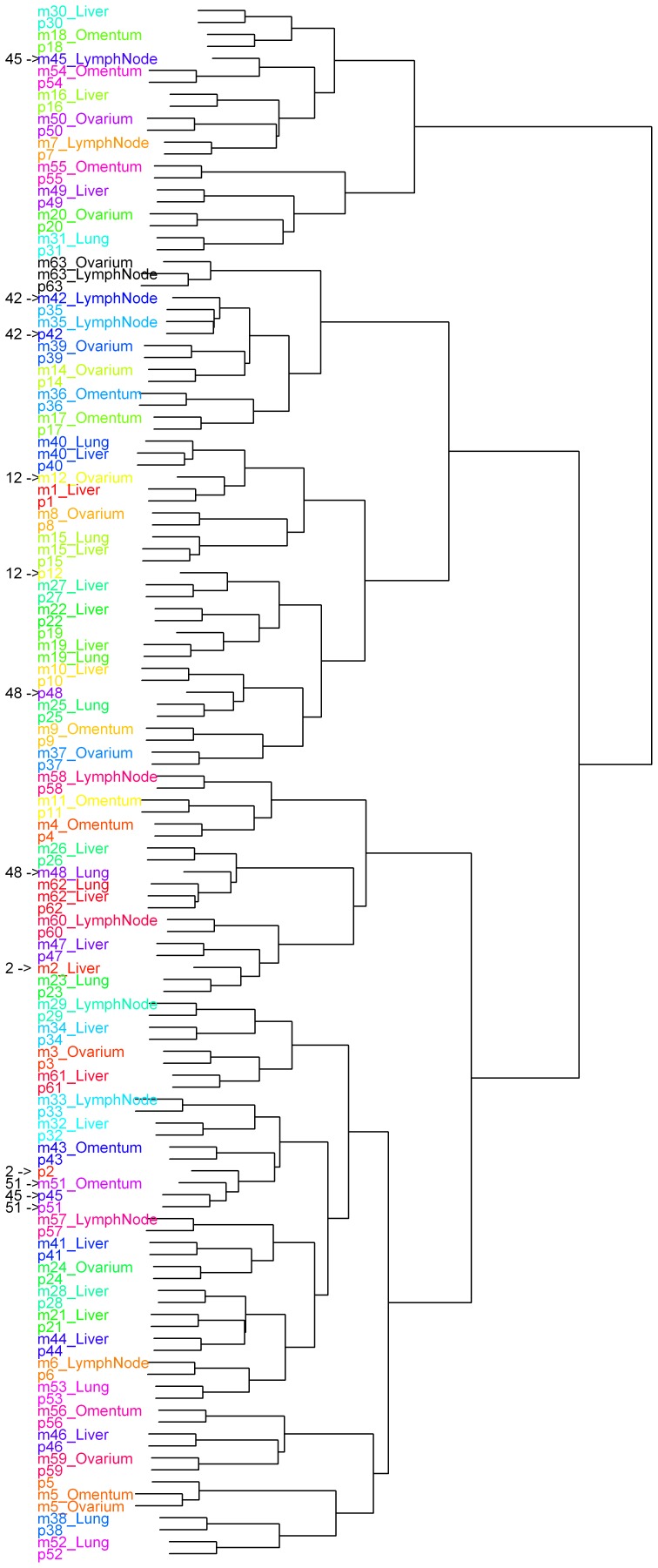
Dendrogram of unsupervised hierarchical clustering of DNA copy number aberrations of all tumors. All tumors includes 62 primary CRC tumors (p) and 68 matched metastases (m). The numbers and pointers on the left show the patients of which the primary tumor and metastasis did not cluster together.

Cluster analysis revealed that DNA copy number profiles of pairs are more similar to each other than between tumors of different patients. For 6 patients, metastases and the corresponding primary tumors were not joined pairwise in the cluster dendrogram (Figure 3). Histological re-evaluation showed similar morphologies within each of these 6 matched pairs. Two of the 6 patients clustered with only one tumor or tumor pair between them. The remaining 4 copy number profiles are shown in Figure S1.

### Non-recurrent Differences between Primaries and Metastases of Individual Patients

**Figure 4:**
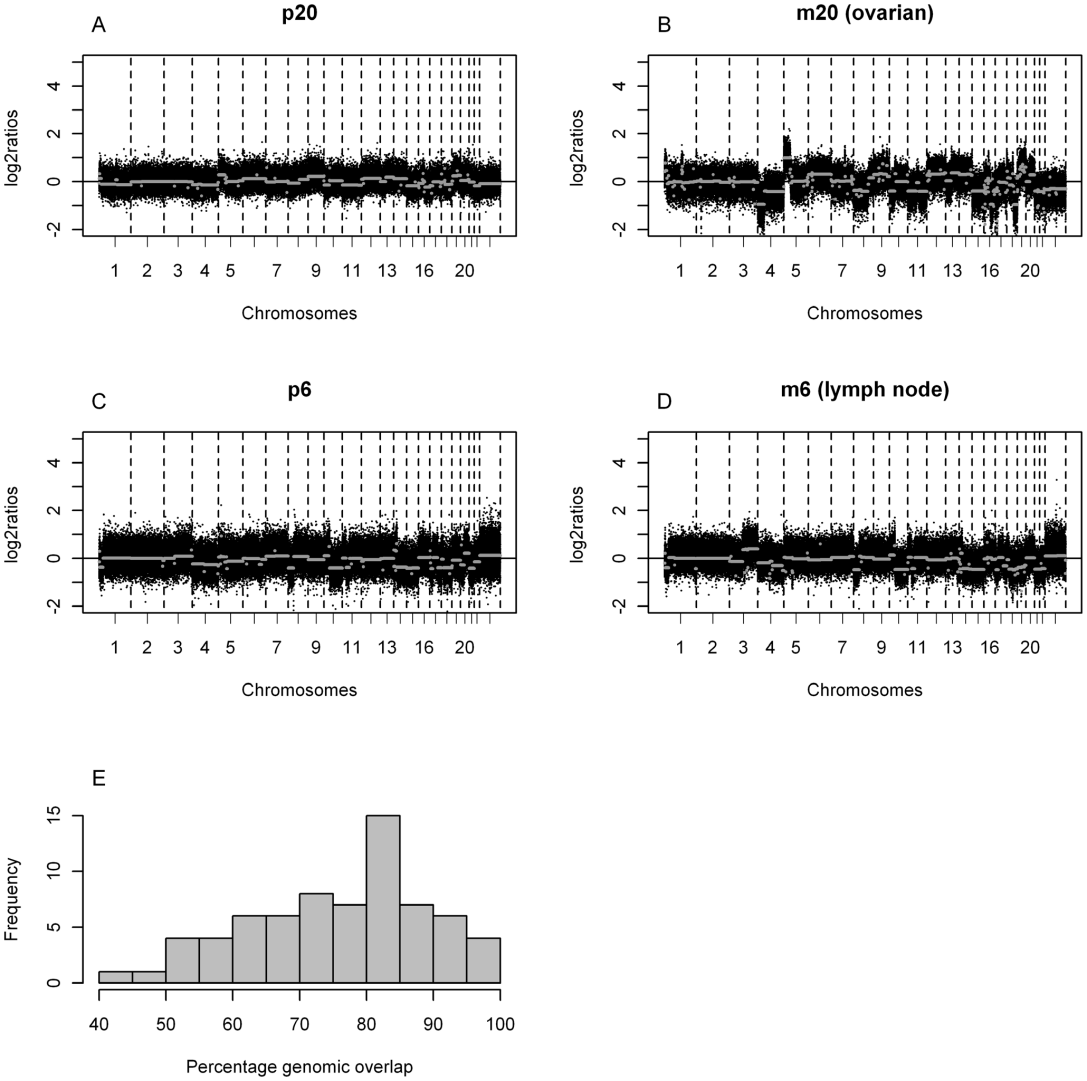
Genomic overlap of primary tumors (p) and matched metastasis (m). (*A–B*) Example of a tumor metastasis pair from the group with less than 70% genomic overlap of gain, loss or normal DNA copy number (total 20 patients of which 3 patients were not joined pairwise in the dendrogram of Figure 3)and (*C–D*) example of a tumor metastasis pair from the group with 70 and 95% genomic overlap of gain, loss or normal DNA copy number (38 patients of which 1 patient was not joined pairwise in the dendrogram of Figure 3) The x-axis displays clones spotted on the array sorted by chromosomal position. The y-axis displays the log_2_ ratios of the clones. The segments are depicted by grey lines. Boundaries of chromosomes are indicated by dotted lines. (*E*) Histogram of the percentages of genomic overlap of gain, loss or normal DNA copy number of all tumor metastasis pairs. The x-axis displays the percentages of overlap. Tthe y-axis displays the frequencies of the metastases.

Overall, a median of 27% of aberrant genome was detected in primary tumors versus a median of 33% in the metastases. This difference is not a consequence of tumor cell percentage which was corrected for. We performed a pair wise comparison of the metastases and the primary tumor per metastasis (Figure 4). This comparison revealed 4 metastases with genomic overlap with the primary tumor of gain, loss or normal DNA copy number of more than 95% (median 96.8% (95.6–100%)). In addition, 43 metastases showed overlap between 70 and 95% (median 82.0% (72.0–94.7%)), and the remaining 21 metasases had an overlap in of less than 70% (median 60.3% (41.6–69.7%)) No specific metastatic site was overrepresented in any of these groups. Three of the four patients that did not join pairwise in the cluster analysis are in the group of less than 70% overlap (53.4, 67.8 and 69.7%). The remaining sample that did not cluster pairwise had an overlap of 82%.

To establish which genomic regions show overall differences in copy number aberrations between the group of primary and metastatic tumors we generated a combined dataset. The log_2_ values of the primary tumors were subtracted from the log_2_ values of the metastases for each position (unique oligonucleotide on the array) by GISTIC [24]. We observed 15 statistically significant events with, 13 regions of lower DNA copy number ratios and 2 regions of higher DNA copy number ratios in the metastases compared to the primary (Table S2). The significant GISTIC peaks identified were due to a high log_2_ ratio of metastases versus primary tumors (high level amplification)rather than a high frequency, hence these are high amplifications in the metastases rather than recurrent gains (or losses). One exception is a co-amplification that we detected in 3 metastases of two patients(see below).

The same approach revealed 7 regions with higher DNA copy number ratio and 8 regions with lower DNA copy number ratio in the 22 liver metastases compared to the primary tumor (Table S3). In the 12 omental metastases 1 region showed a higher copy number ratio and 5 regions lower copy number ratio in comparison to the primary tumor (Table S4). In the other metastatic organs (ovary, lung, and distant lymph nodes) significant differences in DNA copy number ratios between metastases and primary tumors were not observed.

Stange et al. [9], reports a difference at chromosome 11p15.5 in a study of 21 patients with liver metastases. Re-analysis of the data according to our procedures described here, confirms the 11p15.5 gain in 6 liver metastases not present in the primary tumors. On this array CGH platform 3 BAC clones are located within this region. Neither in the 22 patients with liver metastases nor in the remaining 40 patients with other metastatic sites, gain of this region was detected, despite the 38 oligonucleotides located within this chromosomal region. Two out of 62 patients (3%) were MSI (patient 18 and 30). The percentages of genomic overlap of copy number aberrations between the primary tumors and their metastasis in our MSI patients were 88% and 96% respectively. Moreover the primary tumor and the metastases of these patients clustered next to each other. Due to the low incidence of MSI tumors in our study population, this does not affect the conclusions of our study, neither we can conclude anything about this subtype because of low sample size.

### A Co-amplification only Observed in Metastasis Including the *MYC* Oncogene

**Figure 5:**
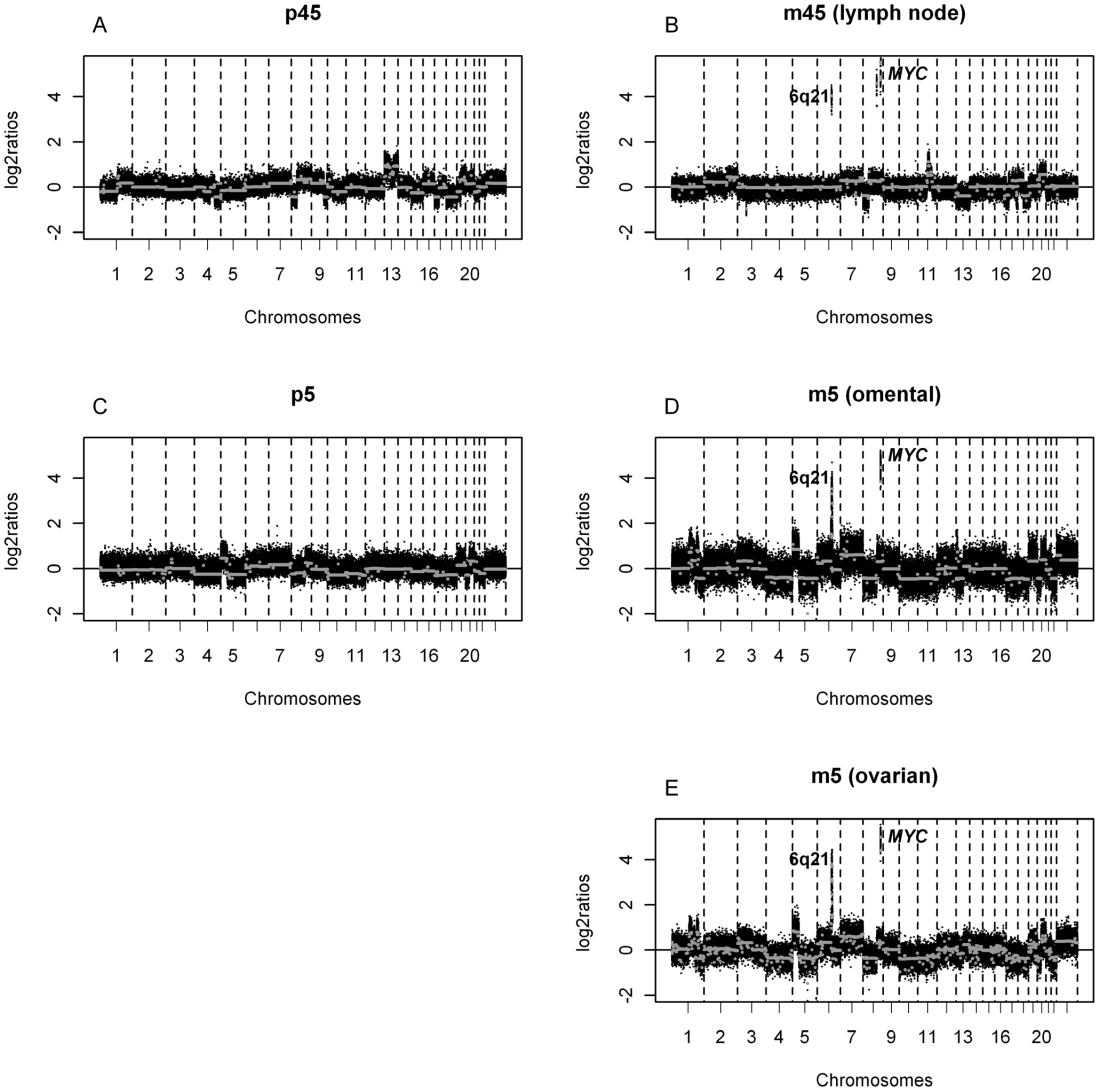
DNA copy number profiles of metastases containing a co-amplification at chromosome 8q24.21 and 6q21. DNA copy number profiles of two patients containing a co-amplification of at 8q24.21 (*MYC*) and chromosome 6q21 in the metastasis (*B, D, E*), which was not present in the primary tumor (*A, C*). The x-axis displays clones spotted on the array sorted by chromosomal position. The y-axis displays the log_2_ ratios of the clones. The segments are depicted by grey lines. Boundaries of chromosomes are indicated by dotted lines.

**Figure 6:**
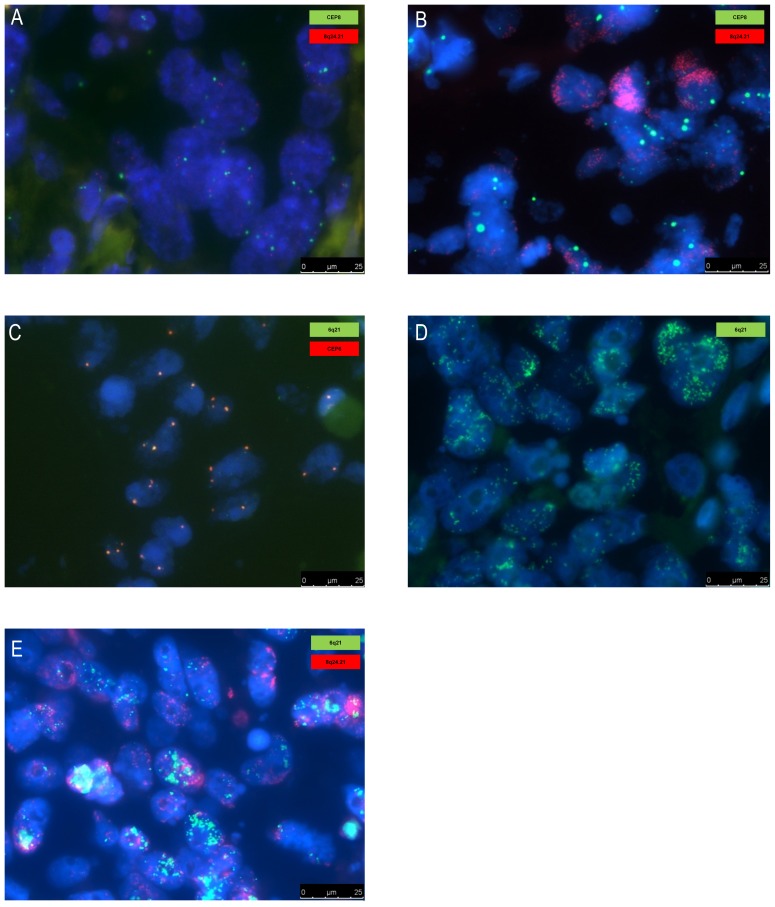
FISH analysis confirming high level co-amplification of *MYC* (8q24.21) and chromosome 6q21. FISH analysis confirming high level co-amplification of *MYC* (8q24.21) and chromosome 6q21 in metastatic tissue (lymph node, sample m45) which were absent in the matched primary tumor (sample p45). (*A*) Primary tumor without the *MYC* amplification (red probe: *MYC* (8q24.21), green probe: centromere chromosome 8). (*B*) Distant lymph node metastasis with the *MYC* (8q24.21) amplification (red probe: *MYC* (8q24.21), green probe: centromere chromosome 8, gain). (*C*) Primary tumor without the chromosome 6q21 amplification (green probe: 6q21, red probe: centromere chromosome 6). (*D*) Distant lymph node metastasis with the 6q21 amplification (green probe: 6q21). (*E*) Co-amplification of *MYC* (8q24.21) and chromosome 6q21 in a distant lymph node (red probe: *MYC*, green probe: 6q21). Abbreviations: CEP6; centromere chromosome 6, CEP8; centromere chromosome 8.

In only two patients we observed a recurrent high level co-amplification in the metastases, which was not detected in the primary tumor. The co-amplification is located at 6q21 and 8q24.21, the latter encompassing the *MYC* oncogene. Respectively 16 and 19 genes are located on these regions listed in Table S2. One of these patients had two metastatic sites involved, both harboring this co-amplification (Figure 5). These array CGH results were confirmed by FISH analysis showing high level amplifications of *MYC* and chromosome 6q21 (Figure 6). The co-amplification did not result from translocation, since no co-localization was observed by FISH. Moreover we did not observe subclones with high level amplification of *MYC* or chromosome 6q21 alone.

We analyzed copy number profiles of 349 primary colorectal tumors of the CAIRO studies and 193 primary colorectal tumors of the Cancer Genome Atlas Network [27]. We detected high level amplification of *MYC*, once in the CAIRO and 3 times in the Atlas datasets. Amplification at 6q21 was once detected only in the CAIRO datasets. The co-amplification was not observed in these 542 primary tumors.

## Discussion

Primary tumors and their metastases are genetically highly similar. Therefore, we reason that many chromosomal aberrations arise in the primary tumor before metastatic spread. In the past years, genetic data have become available that support the idea that the metastatic behavior seems to be predetermined relatively early in tumorigenesis. First, micrometastases are observed in many individuals with small, low-stage tumors [28]. Second, RNA expression profiling of the bulk of primary tumors predict the metastatic recurrence of cancer patients [29], [30]. Third, microarray analysis revealed that RNA expression and DNA copy number patterns of metastatic tumor cells were strikingly similar to that of the primary tumor [9], [10], [31]. Sequence analysis of coding regions in primary and metastatic tumor genomes also suggest that only a few mutations are required to transform cells from an invasive colorectal tumor into cells that have the capability to metastasize [32]. Genome wide sequencing of matched primary and metastatic tissues has only been performed in small patient cohorts. In the study of Kloosterman et al. [33], significant overlap in somatic structural changes between 4 primary tumors and their corresponding metastases was observed. Moreover, whole-genome sequencing of matched primary pancreatic tumors and metastases [34], and genomic analyses of primary prostate cancer and metastases [35] revealed highly similar genomic profiles in these solid malignancies as well. This suggests that essential mutations and chromosomal aberrations required for cancer progression would occur in the primary tumor before initiation of the metastatic spread.

Nevertheless, differences are observed in DNA copy numbers between primaries and metastases, for which several potential scenarios are possible. Either genetic changes occur because the primary and metastasis are different branches from a common yet heterogeneous ancestor [36], or changes occur *after* dissemination. We hypothesize that the most likely scenario is a combination of both heterogeneity within the primary tumor and post-dissemination effects. It is thereby important to take into account that chromosomal aberrations in less than 30% of tumor cells can go undetected with arrays [37]. Since a primary colorectal tumor can be quite large and only a small cross section was taken for copy number analysis, heterogeneity would be reflected in the copy number measurements and explain some of the differences between matched primaries and metastases. Another explanation for the observed differences could be that the studied metastasis arose from a distinct primary. Some of the patients included in our cohort presented with metachronous metastases and consequently received (neo)adjuvant systemic treatment. The effect of systemic treatment on chromosomal instability however is largely unknown, but probably limited since no recurrences were identified and no significantly increased number of gene variants (associated with pathways relevant in cancer) were observed as a result of chemotherapy [38]. Our data strengthen this observation because the patients who did receive (neo)adjuvant systemic treatment clustered pairwise. None of the 6 patients who did not cluster together received chemotherapy and targeted agents.

Stange et al. [9] published a dataset of 21 paired samples where a characteristic gain was found in 6 of 21 liver metastases (29%) on chromosome band 11p15.5 that was observed in only 1 primary tumor (5%). They confirm this observation in an independent dataset of liver metastases (n = 50, 30% gain). The Cancer Genome Atlas Network recently reported that one of the most common focal gains is at 11p15.5 in 7% of primary CRC tumors [27]. In our dataset we observe ca. 10% gain of the same region without a significant difference between both primary and metastasis (Figure 2). Thus although we could confirm a same frequency of gain in the primary tumors, we were not able to confirm a higher frequency in our set of patients with liver metastases (n = 22), nor in other metastatic sites. We conclude that (focal) gain at 11p15.5 is not prevalent for metastasis in our dataset, as opposed to the reported findings by Stange et al. [9]. We only observed one recurrent event; two patients with co-amplifications on the same chromosomal locations in the metastases, which were not present in the primary tumor. This co-amplification was not detected previously in larger series of primary tumors, nor in smaller studies of metastases. Previous series of metastases did not use high resolution array CGH, and may thus have missed this focal co-amplification. Hence, the association of this specific co-amplification with metastasis needs further confirmation by high resolution copy number analysis on a large series of CRC metastasis. In the era of personalized anti-cancer treatment it is essential to understand the diverging features between primaries and metastases and which tissue will best predict treatment outcome. Current clinical practice is to use archived material of the primary tumor to determine molecular aberrations and mutations to select patients for treatment, whereas in fact therapy is oriented towards treating the metastases. Since genomic profiles are highly similar between the primary tumor and the metastasis this approach is now justified and it is unlikely that precursor cells of overt metastases in CRC disseminate early to sites where they proceed to undergo their own divergent genetic evolution.

### Array Data Availability

GEO accession number GSE38479.

Token:

http://www.ncbi.nlm.nih.gov/geo/query/acc.cgi?token=fjaflewyayqeapw&acc=GSE38479.

## Supporting Information

Figure S1 **DNA copy number profiles.** DNA copy number profiles of patients of which the correlation of the primary tumor (p) and their metastasis (m) was substantially low with more than one tumor pair between them. The patients showed genomic overlap of gain, loss or normal DNA copy number of (*A–B*) 67.8%, (*C–D*) 82.0%, (*E–F*) 69.7% and (*G–H*) 53.4%. The x-axis displays clones spotted on the array sorted by chromosomal position. The y-axis displays the log_2_ ratios of the clones. The segments are depicted by grey lines. Boundaries of chromosomes are indicated by dotted lines.
(TIFF)

Table S1 **Comparison of frequencies of DNA copy number aberrations between the primary tumors and the metastases.** Abbreviations: FDR; false discovery rate.
(DOC)

Table S2 **GISTIC approach in combined samples.** *Regions which overlap with the results of liver metastasis, ** Regions which overlap with the results of omental metastases. Abbreviations: FDR; false discovery rate.
(DOC)

Table S3 **GISTIC approach in combined samples of liver metastasis.** Abbreviations: FDR; false discovery rate.
(DOC)

Table S4 **GISTIC approach in combined samples of omental metastasis.** Abbreviations: FDR; false discovery rate.
(DOC)
