# Supplementary figures and images for: Chromosomal Copy Number Aberrations in Colorectal Metastases Resemble Their Primary Counterparts and Differences Are Typically Non-Recurrent
Source: PLoS One. 2014 Feb 5;9(2):e86833. doi: 10.1371/journal.pone.0086833 (PMC3914793; doi:10.1371/journal.pone.0086833)

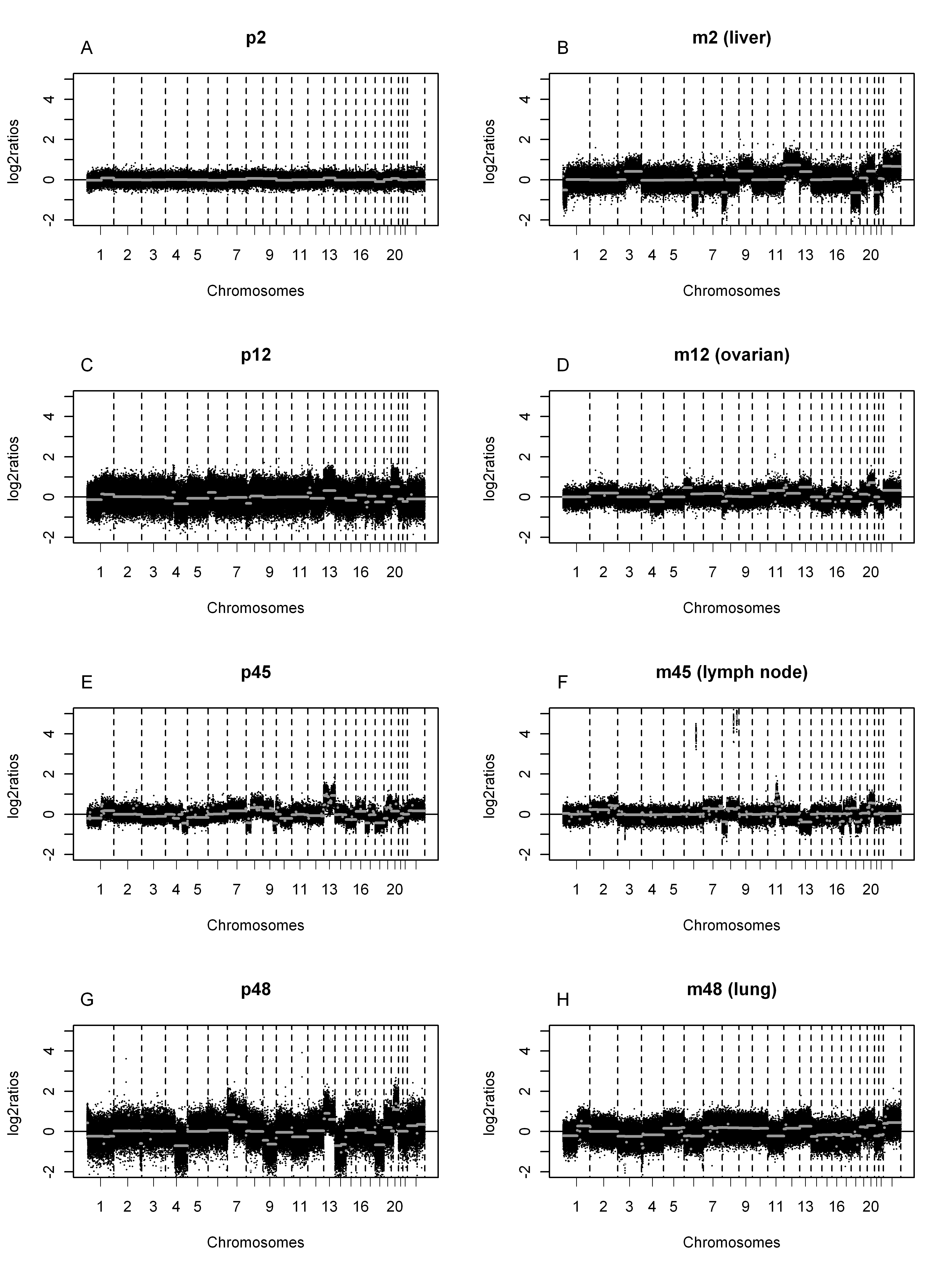

Supplement: Figure S1 — DNA copy number profiles. DNA copy number profiles of patients of which the correlation of the primary tumor (p) and their metastasis (m) was substantially low with more than one tumor pair between them. The patients showed genomic overlap of gain, loss or normal DNA copy number of (A–B) 67.8%, (C–D) 82.0%, (E–F) 69.7% and (G–H) 53.4%. The x-axis displays clones spotted on the array sorted by chromosomal position. The y-axis displays the log2 ratios of the clones. The segments are depicted by grey lines. Boundaries of chromosomes are indicated by dotted lines. (TIFF) [file pone.0086833.s001.tiff]
