# Supplementary material for: Chromosomal Copy Number Aberrations in Colorectal Metastases Resemble Their Primary Counterparts and Differences Are Typically Non-Recurrent
Source: PLoS One. 2014 Feb 5;9(2):e86833. doi: 10.1371/journal.pone.0086833 (PMC3914793; doi:10.1371/journal.pone.0086833)
Supplement: Table S1 — Comparison of frequencies of DNA copy number aberrations between the primary tumors and the metastases. Abbreviations: FDR; false discovery rate. (DOC) [file pone.0086833.s002.doc]

**Table S1. Comparison of frequencies of DNA copy number aberrations between the primary tumors and the metastases.**

| Region | Band | Copy number status | Frequency in metastasis (%) | Frequency in primary tumour (%) | p-value | FDR |
| --- | --- | --- | --- | --- | --- | --- |
| chr2:2,609,073-2,646,266 | 2p25.3 | Gain | 36.8 | 8.1 | < 0.001 | 0.42 |
| chr2:2,646,266-2,768,821 | 2p25.3 | Gain | 38.2 | 8.1 | < 0.001 | 0.42 |
| chr2:2,786,596-3,216,115 | 2p25.3 | Gain | 39.7 | 9.7 | < 0.001 | 0.42 |
| chr2:2,768,821-2,786,596 | 2p25.3 | Gain | 38.2 | 9.7 | < 0.001 | 0.45 |
| chr2:3,216,115-3,233,076 | 2p25.3 | Gain | 35.3 | 9.7 | < 0.001 | 0.45 |
| chr2:3,233,076-3,250,127 | 2p25.3 | Gain | 35.3 | 8.1 | < 0.001 | 0.45 |
| chr2:3,250,127-3,281,460 | 2p25.3 | Gain | 33.8 | 8.1 | < 0.001 | 0.45 |
| chr2:3,281,460-3,312,704 | 2p25.3 | Gain | 32.4 | 6.5 | < 0.001 | 0.45 |
