# Supplementary material for: Chromosomal Copy Number Aberrations in Colorectal Metastases Resemble Their Primary Counterparts and Differences Are Typically Non-Recurrent
Source: PLoS One. 2014 Feb 5;9(2):e86833. doi: 10.1371/journal.pone.0086833 (PMC3914793; doi:10.1371/journal.pone.0086833)
Supplement: Table S2 — GISTIC approach in combined samples. *Regions which overlap with the results of liver metastasis, ** Regions which overlap with the results of omental metastases. Abbreviations: FDR; false discovery rate. (DOC) [file pone.0086833.s003.doc]

**Table S2**. **GISTIC approach in combined samples**

| Extended Region | Band | Type | FDR | G-Score | Genes |
| --- | --- | --- | --- | --- | --- |
| chr1:1,438,247-12,034,621* | 1p36.33-p36.22 | Loss | 0.002 | 8.1 | >50 genes |
| chr3:60,181,256-60,563,627 | 3p14.2 | Loss | 0.005 | 7.8 | FHIT |
| chr4:4,626,306-31,693,271 | 4p16.2-p15.1 | Loss | 0.05 | 6.8 | >50 genes |
| chr4:85,226,936-92,648,432 | 4q21.23-q22.1 | Loss | 0.006 | 7.7 | >50 genes |
| chr6:105,350,190-107,821,131 | 6q21 | Gain | < 0.001 | 12.6 | HACE1, LIN28B, BVES, C6orf112, POP3, POPDC3, PREP, PRDM1, ATG5, AIM1, RTN4IP1, QRSL1, AK124400, K025967, LOC100422737, LOC553137, C6orf203, BEND3, PDSS2 |
| chr6:162,581,750-163,143,342 | 6q26 | Loss | 0.006 | 7.7 | Parkin, PARK2, PACRG |
| chr8:39,380,297-39,805,805* | 8p11.23-p11.22 | Loss | < 0.001 | 13.4 | ADAM5P, tMDC, ADAM3A, tMDC III, ADAM18, ADAM2 |
| chr8:127,000,942-129,605,179** | 8q24.13-q24.21 | Gain | < 0.001 | 18.8 | LOC100130231, BX648371, FAM84B, K125310, BC106081, DQ515898, DQ515899, DQ515897, POU5F1B, POU5F1, LOC727677, BC042052, MYC, MIR1204, TMEM75, PVT1, MIR1205, MIR1206, MIR1207, MIR1208, BC009730 |
| chr16:6,458,969-6,559,580* | 16p13.2 | Loss | 0.001 | 8.4 | A2BP1, RBFOX1 |
| chr18:3,458,748-13,680,560 | 18p11.31-p11.21 | Loss | < 0.001 | 8.8 | >50 genes |
| chr18:48,738,234-57,604,348* | 18q21.2-q21.33 | Loss | < 0.001 | 10.8 | >50 genes |
| chr20:14,932,222-15,035,264** | 20p12.2 | Loss | < 0.001 | 9.9 | MACROD2 |
| chr21:35,205,722-35,651,123* | 21q22.12 | Loss | 0.003 | 8 | C21orf96, RUNX1 |
| chrX:7,022,874-7,471,041** | Xp22.31 | Loss | < 0.001 | 11.2 | HDHD1A, HDHD1, MIR4767, STS |
| chrX:88,378,677-88,554,287** | Xq21.31 | Loss | 0.002 | 8.2 | No genes |
