# Supplementary material for: Chromosomal Copy Number Aberrations in Colorectal Metastases Resemble Their Primary Counterparts and Differences Are Typically Non-Recurrent
Source: PLoS One. 2014 Feb 5;9(2):e86833. doi: 10.1371/journal.pone.0086833 (PMC3914793; doi:10.1371/journal.pone.0086833)
Supplement: Table S3 — GISTIC approach in combined samples of liver metastasis. Abbreviations: FDR; false discovery rate. (DOC) [file pone.0086833.s004.doc]

**Table S3**. **GISTIC approach in combined samples of liver metastasis.**

| Extended Region | Band | Type | FDR | G-Score | Genes |
| --- | --- | --- | --- | --- | --- |
| chr1:1,438,247-12,034,621 | 1p36.33-p36.22 | Loss | 0.004 | 4.6 | >50 genes |
| chr1:14,422,773-39,754,316 | 1p36.21-p34.2 | Loss | 0.033 | 3.8 | >50 genes |
| chr1:147,449,399-188,244,161 | 1p36.21-p34.2 | Gain | 0.021 | 3 | >50 genes |
| chr6:38,597,304-47,734,508 | 6p21.2-p12.3 | Gain | 0.05 | 2.8 | >50 genes |
| chr8:22,872,222-43,647,122 | 8p21.3-p11.1 | Loss | 0.003 | 6.2 | >50 genes |
| chr13:73,188,285-114,142,980 | 13q22.1-q34 | Gain | 0.011 | 3.3 | >50 genes |
| chr15:59,213,849-69,545,245 | 15q22.2-q23 | Loss | 0.026 | 3.9 | >50 genes |
| chr16:6,458,969-6,611,810 | 16p13.2 | Loss | 0.006 | 4.4 | A2BP1 |
| chr18:39,210,072-76,117,153 | 18q12.3-q23 | Loss | 0.029 | 3.8 | >50 genes |
| chr20:0-13,973,599 | 20p13-p12.1 | Gain | 0.009 | 3.8 | >50 genes |
| chr20:29,297,270-62,435,964 | 20q11.21-q13.33 | Loss | 0.042 | 3.7 | >50 genes |
| chr20:56,903,488-62,435,964 | 20q13.32-q13.33 | Gain | 0.009 | 3.9 | >50 genes |
| chr21:35,171,630-41,168,771 | 21q22.12-q22.2 | Loss | 0.017 | 4 | >50 genes |
| chrX:23,845,804-54,782,977 | Xp22.11-p11.21 | Gain | 0.005 | 4.5 | >50 genes |
| chrX:92,221,241-96,621,273 | Xq21.32-q21.33 | Gain | 0.005 | 4.5 | NAP1L3, FAM133A, LOC643486, DIAPH2, RPA4 |
