# Supplementary material for: Chromosomal Copy Number Aberrations in Colorectal Metastases Resemble Their Primary Counterparts and Differences Are Typically Non-Recurrent
Source: PLoS One. 2014 Feb 5;9(2):e86833. doi: 10.1371/journal.pone.0086833 (PMC3914793; doi:10.1371/journal.pone.0086833)
Supplement: Table S4 — GISTIC approach in combined samples of omental metastasis. Abbreviations: FDR; false discovery rate. (DOC) [file pone.0086833.s005.doc]

**Table S4**. **GISTIC approach in combined samples of omental metastasis.**

| Extended Region | Band | Type | FDR | G-Score | Genes |
| --- | --- | --- | --- | --- | --- |
| chr8:0-36,814,774 | 8p23.3-p12 | Loss | 0.043 | 2.8 | >50 genes |
| chr8:126,255,874-146,274,826 | 8q24.13-q24.3 | Gain | 0.001 | 6 | >50 genes |
| chr18:18,179,005-76,117,153 | 8q11.2-q23 | Loss | 0.043 | 2.7 | >50 genes |
| chr20:0-25,591,221 | 20p13-p11.21 | Loss | 0.043 | 2.6 | >50 genes |
| chrX:7,022,874-49,560,627 | Xp22.31-p11.23 | Loss | 0.043 | 3.1 | >50 genes |
| chrX:61,848,414-94,008,535 | Xq11.1-q21.33 | Loss | 0.043 | 2.5 | >50 genes |
